# Diffuse Midline Gliomas: Challenges and New Strategies in a Changing Clinical Landscape

**DOI:** 10.3390/cancers16010219

**Published:** 2024-01-02

**Authors:** Umberto Tosi, Mark Souweidane

**Affiliations:** 1 Department of Neurological Surgery, Weill Cornell Medicine, New York, NY 10021, USA; 2 Department of Neurological Surgery, Memorial Sloan Kettering Cancer Center, New York, NY 10065, USA

**Keywords:** diffuse midline, glioma, glioblastoma, midline, thalamus

## Abstract

**Simple Summary:**

Diffuse midline gliomas are rare tumors of childhood characterized by a midline location, a diffuse-infiltrative growth pattern, and newly described genetic and epigenetic mutations. Since their first description at the beginning of the 20th century, they have been inoperable and carried a poor prognosis, with median survival shorter than two years. In the last decade, however, new hope is on the horizon thanks to new understanding of these tumors’ biology and the development of novel, targeted therapeutics.

**Abstract:**

Diffuse intrinsic pontine glioma (DIPG) was first described by Harvey Cushing, the father of modern neurosurgery, a century ago. Since then, the classification of this tumor changed significantly, as it is now part of the broader family of diffuse midline gliomas (DMGs), a heterogeneous group of tumors of midline structures encompassing the entire rostro-caudal space, from the thalamus to the spinal cord. DMGs are characterized by various epigenetic events that lead to chromatin remodeling similarities, as two decades of studies made possible by increased tissue availability showed. This new understanding of tumor (epi)biology is now driving novel clinical trials that rely on targeted agents, with finally real hopes for a change in an otherwise unforgiving prognosis. This biological discovery is being paralleled with equally exciting work in therapeutic drug delivery. Invasive and noninvasive platforms have been central to early phase clinical trials with a promising safety track record and anecdotal benefits in outcome.

## 1. A Moving Target

One of the first historical descriptions of diffuse midline gliomas is attributed to Harvey Cushing, the father of modern neurosurgery, who was challenged with the case of a 15-year-old girl presenting long tract signs and cranial neuropathies. Following an unsuccessful exploratory suboccipital craniectomy (lateral enough to approach the mastoid process), augmented by a cerebellar hemispherectomy, Cushing himself had to raise the white flag as the tumor, what was later defined as a diffuse intrinsic pontine glioma (DIPG), was not amenable to surgical resection given its inseparability from the high-stake cytoarchitecture of the pontine nuclei [1,2,3].

Over the following century, the prognosis and treatment approach for DIPG and other diffuse midline gliomas (DMGs) remained virtually unchanged, with these tumors not being amenable to surgical resection. Similarly, despite astounding leap forwards in other liquid and solid malignancies, no effective chemotherapy to date has been developed for DMGs. External beam radiation therapy is now a mainstay of (palliative) treatment [4,5].

Over the last two decades, therapeutic efforts aimed at altering DIPG’s dismal prognosis were paralleled by equally herculean endeavors aimed at understanding its biology. Numerous studies were made possible thanks to the increased availability of tissue samples that followed a philosophical change: DIPG’s biopsies, though they could not lead to a change in prognosis, were necessary to better understand these tumors and, since they were deemed safe in a majority of cases, became part of clinical practice again, following their abandonment in the 1990s, when the prospective of a cure was so remote to make the risks associated with tissue sampling unjustifiable [6,7]. This culminated in the recognition, across 1000 samples of pediatric high-grade gliomas, of key mutations believed to hold therapeutic potential: H3.3G34R, H3.3K27M, and H3.1K27M [8].

A new diagnosis of DIPG and other DMGs is thus in order. DMGs are glial-based tumors of midline structures (thalami, pontine trunk, spine) characterized by glial features and a diffuse-infiltrative growth pattern. The exact pathological definition of these entities changed significantly between 2016 (when the fourth edition of the WHO Classification of Tumors of the Central Nervous System was adapted) and 2021, when its fifth edition came into effect [9,10]. In 2016, recognizing numerous biopsy studies that identified H3.3K27M as an essential mutation in the genome of diffuse intrinsic pontine glioma (DIPG), a tumor thus far diagnosed mainly based on magnetic resonance imaging (MRI) characteristics, led to a mutation-based diagnosis: such an entity was defined as a “DMG, H3K27M-mutant.” As such, this mutation was sufficient to characterize DIPG as a grade IV DMG, regardless of histological characteristics (the gold standard up to that point). In 2021, this definition was expanded to include other alterations of histone 3 to “DMG H3K27-altered,” whereby other mutations that lead to chromatin remodeling alterations (e.g., loss of H3K27 trimethylation) can be included under the DMG umbrella [11]. In a majority of samples, the H3K27M alterations are due to mutations in two key histone genes: *HIST1H3B/C* (H3.1) and *H3F3A* (H3.3) [12].

Further studies are now trying to segment DMG location and associated mutations; albeit the literature is in need of larger longitudinal studies, new research has emerged indicating how certain anatomical locations have a stronger association with specific mutations (e.g., thalamus with *TP53* or *ATRX*; and pons with *ACVR*). Similarly, mutations are now being associated with gender (e.g., *ACVR* with the female gender) and with outcome (e.g., *TP53* yielding lower survival and *ACVR* longer) [12]. Noticeably, given the new WHO definition of pediatric DMGs, prior work where survival was associated with different genetic and histone alterations (e.g., [8]) has to be reanalyzed in light of these new molecular histopathological entities. Identification of genetic prognostic factors that go beyond the presence/absence of histone 3 mutations is similarly underway and made possible by large collaborative efforts of much-needed statistical power. For instance, analyses of long-term survivors identified MAPK pathway alterations, which now carry a new prognostic value [13].

## 2. Symptomatology and Presentation

**Figure 1:**
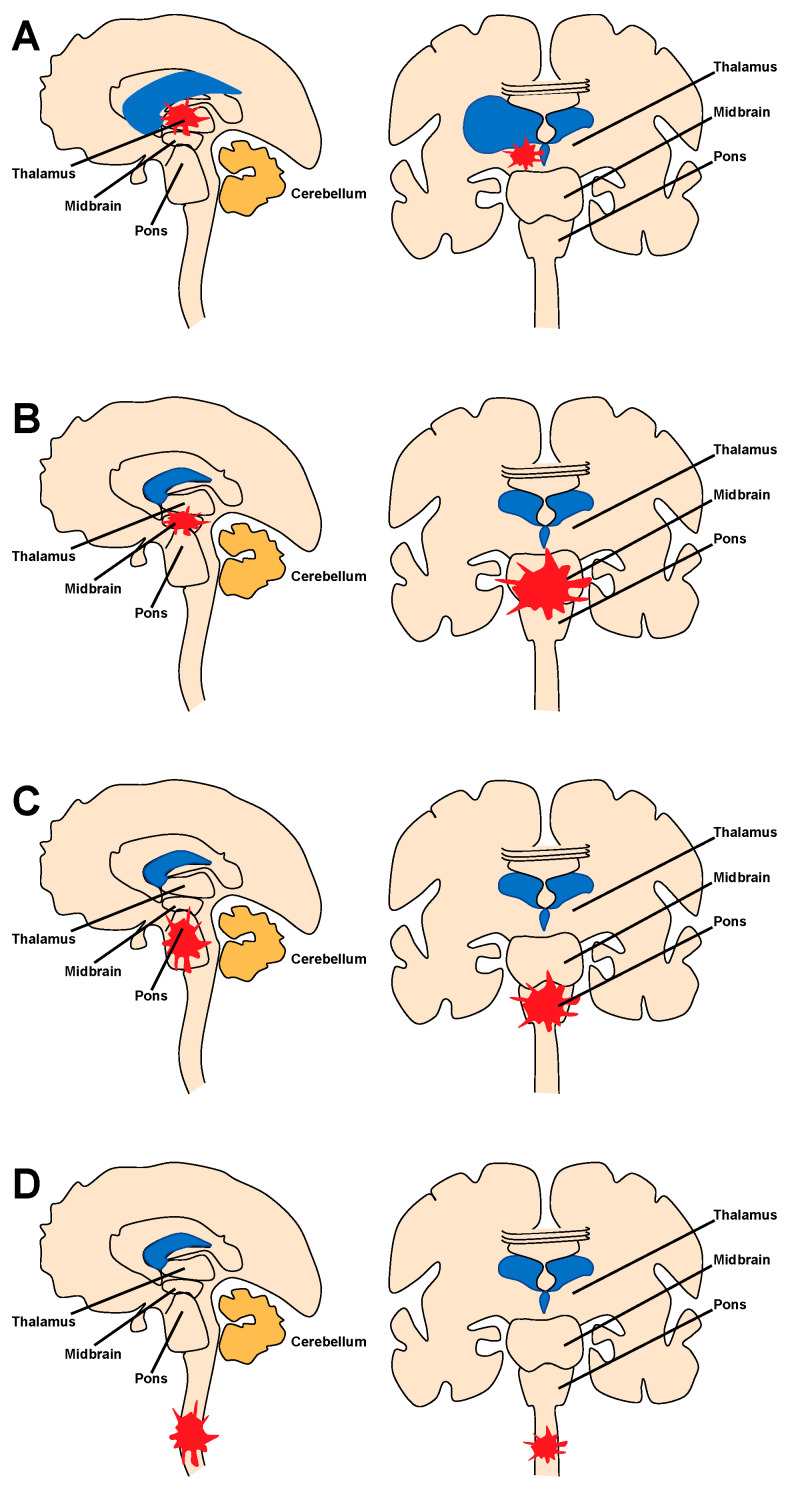
DMGs Location. Sagittal and coronal illustrations showing: (**A**) Thalamic glioma (red) can cause obstructive hydrocephalus with resultant ventriculomegaly (blue). (**B**) Midbrain gliomas can cause tectal symptomatology. (**C**) Pontine gliomas (e.g., DIPG) can present with long-tract signs, cranial neuropathies, and cerebellar symptoms. (**D**) Spinal gliomas can present with symptomatology caudal to the lesion.

DMGs are the second most common malignant pediatric brain tumor, occurring mainly in children 3–10 years of age, with an estimated annual incidence of 200–300 cases per year in the US [14,15]. Symptomatology for DMGs is heavily dependent on location. For instance, thalamic DMGs can present with (contralateral) hemiparesis or hemianesthesia owing to the involvement of motor or sensory thalamic nuclei and potential mass effect on the adjacent internal capsule, as well as obstructive hydrocephalus symptoms (e.g., headache, somnolence) owing to mass effect on the third ventricle (Figure 1A) [16]. On the other hand, midbrain DMGs can present with Perinaud-like syndromes due to tectal and pineal-region involvement (e.g., upward gaze palsy, convergence-retraction nystagmus, and pupillary-light near dissociation), more commonly seen with more benign pineal lesions (Figure 1B) [17,18]. DIPGs, owing to their proximity to pontine nuclei and descending corticospinal tracts, can present with a triad of long-tract signs (e.g., myelopathy), cerebellar symptoms (e.g., ataxia or dysmetria), and cranial neuropathies (often starting as diplopia on lateral gaze) (Figure 1C) [1]. Spinal DMGs often present with symptoms associated with their location, with symptomatic myelopathy caudal to the level of the lesion (e.g., predominant leg symptoms for thoracic lesion) (Figure 1D) [19].

**Figure 2:**
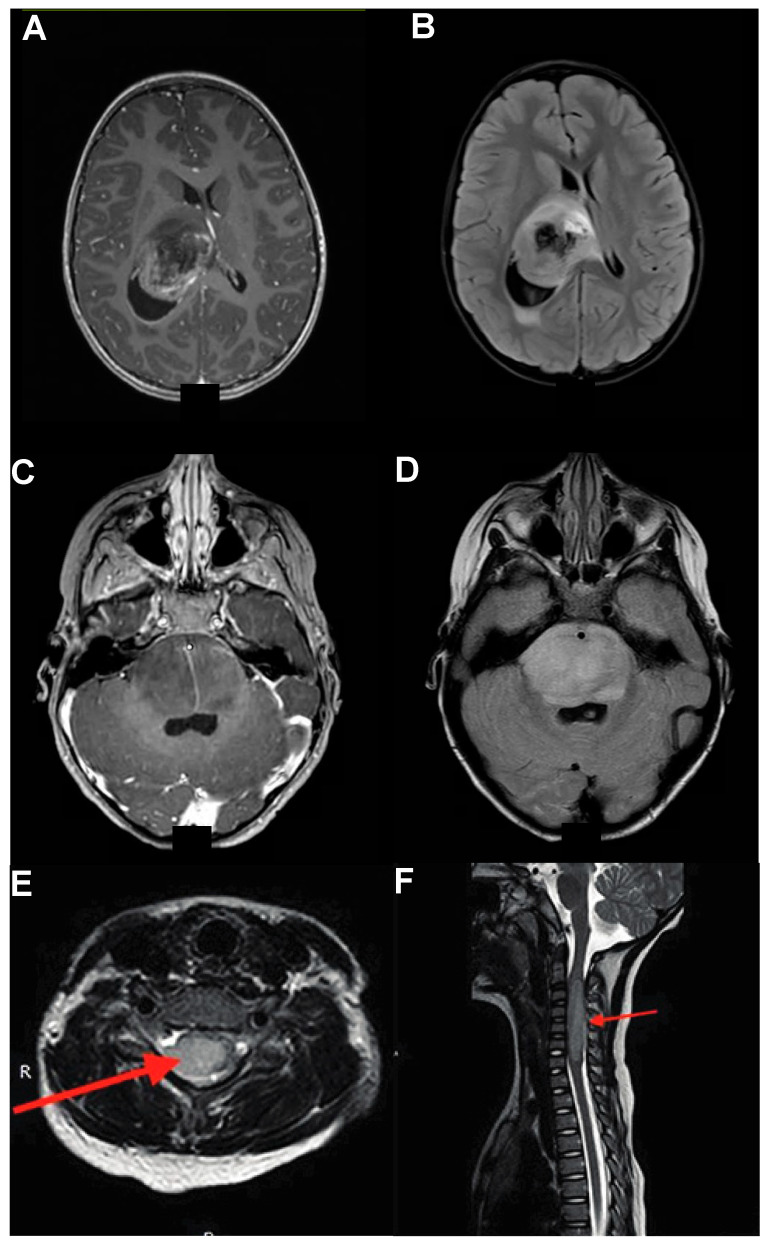
DMGs Imaging Characteristics. (**A**,**B**) Thalamic gliomas show involvement of various thalamic nuclei (**A**) and a significant FLAIR signal (**B**) causing locoregional mass effect, ventricular entrapment, and internal capsule involvement. (**C**,**D**) DIPG and midbrain DMGs are characterized by wispy heterogeneous contrast enhancement (**C**) and enlargement of the pons (**D**). (**E**,**F**) Spinal gliomas, like their cranial counterparts, are characterized by heterogenous contrast enhancement (**E**), with significant cranio-caudal spread (**F**). Adapted from [31].

Given the wide variety of anatomical locations and presentations, it logically follows that DMGs have a complex appearance on MRI, the main modality of imaging (CT, owing to its lower tissue resolution, is not part of standard clinical practice except as a first-line screening tool). Prior to biopsy, a presumptive diagnosis is usually made, then confirmed with tissue sampling, given imaging features different from other pathologies often found in these locations [1,20,21]. It is important to note how DMGs are famous for their occasionally heterogenous radiographic and MRI appearances, with more than one expert practitioner challenged by unusual imaging characteristics. At the same time, separating DMGs from other clinical entities remains essential, given the diametrically opposed treatment algorithms available for other tumor types. With this being said, in general, thalamic gliomas usually present as a thalamic-centered lesion (unilateral or bilateral); contrast enhancement is common but not universal, heterogenous, and not well circumscribed, unlike other low-grade gliomas (e.g., pilocytic, not H3.3K27M mutated) (Figure 2A). Locoregional mass effect on the internal capsule or third or lateral ventricle, diffusion restriction on diffusion-weighed imaging, and tumor-associated edema (seen as FLAIR signal) are often seen as well (Figure 2B) [19,22,23].

Midbrain DMGs rest on the same spectrum as DIPGs: in cases of infiltrative lesions, the border between the midbrain and pons is often blurred, with lesions crossing the superior pontine sulcus with an inferior trajectory (if arising in the midbrain proper) or a superior one (if from the pons). Radiographically, these tumors share common features, such as an enlargement of the native cytoarchitecture (for DIPG, upwards of 50% of the pons), a wispy, non-capsular gadolinium enhancement, and a significant FLAIR signal that often extends beyond the contrast-enhancement pattern (Figure 2C,D) [24,25]. Importantly, dichotomizing FLAIR signal into tumor proper and reactive inflammation without obvious tumor cells has been a challenge and the matter of much research aimed at defining better biopsy targets [26,27]. Albeit the subject of much research, this matter is yet to be put to rest: novel imaging modalities are emerging in an effort to answer this question, but to date, biopsy can be the only definitive diagnostic. Importantly, DMGs can be differentiated from other (more benign) pontine lesions, such as tectal gliomas and dorsal exophytic tumors. Tectal gliomas tend to have a growth pattern restricted to the tectal plate, without contrast enhancement; dorsal exophytic tumors, as the name suggests, present as exophytic growths from the pontine trunk, with a clearly demarcated border separating them from the brainstem.

Spinal DMGs share features with their cranial counterparts, with growth patterns logically restricted by the limited axial spread possible within the spinal cord; thus, these lesions have a predominant rostro-caudal elongation rather than anterior–posterior or medio-lateral one. They are characterized by a significant FLAIR signal often spanning multiple spinal levels and poorly differentiated contrast enhancement (Figure 2E,F) [28,29]. Spinal DMGs can be difficult to distinguish from other astrocytic spinal lesions; these tend to be more eccentric and can have stronger contrast enhancement, albeit the two entities can be difficult to separate by MRI alone [30].

Differentiating DMGs from other (usually more benign) pathologies on the ground of MRI alone remains challenging, albeit with significant clinical yield, as other entities (e.g., pilocytic astrocytomas) can be amenable to curative surgical resection. Generally speaking, benign tumors have a more homogeneous contrast enhancement pattern or no enhancement at all, can be accompanied by large fluid-filled cysts, and often have a recognized margin with normal brain parenchyma (either as a CSF-filled arachnoid rim or a subtler pial margin). Similarly, separating H3-3K27M-mutated DMGs from wildtype ones carries significant promise. Novel imaging algorithms based on diffusion and spectral imaging are now being paired with machine learning technologies in the hope of obtaining this goal [23,32]. While these technologies are being developed, however, stereotactic biopsies remain necessary to answer these important questions.

## 3. Historical Failures: In Need of a New Paradigm

Following Cushing’s initial discovery of the inoperability of DIPG owing to its invasion of key brainstem nuclei, surgical interventions for DMGs were rapidly abandoned. Even the need for surgical biopsies was questioned as early as the 1950s, when tissue availability could lead to little to no clinical improvement at a time when roentogen therapy, however, was recognized for its palliative role [33]. The sentiment remained unchallenged until the 1980s, before the time when MRIs were readily available, when early studies showed that the risks associated with biopsy were low and prognostic information could be obtained, namely how a higher number of mitotic figures was associated with worse prognosis [34]. The advent of MRI, however, changed this paradigm, as it became clear that imaging was sufficient for obtaining prognostic information, with diffuse infiltrative tumors of the pons (comparable to today’s DIPG) associated with a worse prognosis than tumors in the midbrain, medulla, or dorsal exophytic pontine tumors [35]. The differences between these tumors and DIPGs were striking: dorsal exophytic tumors, for instance, had a characteristic growth pattern and typical contrast enhancement pattern that made them easily recognizable by MRI. They were considered benign and amenable to surgical resection, which could be curative. This was due, in part, to a more benign histology (e.g., pilocytic) [36,37,38,39]. The ability to separate such benign histology (e.g., pilocytic) as seen on imaging, regardless of location, from more malignant counterparts allowed for a different surgical approach, as gross total resection can often be curative for well-encapsulated pilocytic malignancies of both the supratentorial and infratentorial compartments [40]. MRI further allowed for the differentiation between (malignant) pontine DMGs from (benign) tectal lesions, which, albeit carry significant clinical risk in the setting of aqueductal obstruction, often have a benign histology that only warrants CSF diversion in the form of shunting or third ventriculostomy [41,42,43].

Furthermore, a large study where open and stereotactic biopsies were assessed, albeit finding an overall low surgical morbidity, also found no discernable clinical benefit following intervention, as patients underwent radiation therapy similarly to those for whom tissue sampling did not occur, with similar clinical outcomes [44]. Between the 1980s and 1990s, a change then occurred: biopsies, initially believed to be safe and useful for DMGs like they were for other CNS malignancies, were found to offer no clinical benefit and, thus, to carry a useless risk.

With surgery out of the question, the question of whether tissue sampling was needed was paralleled by lukewarm clinical progress. External beam radiation therapy remained the standard of care, with various studies assessing different radiation paradigms without finding significant clinical differences. As summarized in 2006 by a large review, this was paralleled by numerous studies where adjuvant or neoadjuvant systemic chemotherapy was shown to have minimal, if any, clinical benefit with, at most, partial response. No significant changes in overall survival or progression-free survival were observed [45]. It is important to note how, by the mid-2000s, most trials relied on classic chemotherapeutic agents (e.g., topoisomerase inhibitors, platinum-based chemotherapy, vinca alkaloids, etc.) and were stopped early due to systemic side effects that prevented therapeutic dose escalations [46].

The sentiment around biopsy changed around 2012, when it was first shown that stereotactic biopsies were safe and obtained a sufficient amount of tissue for genetic studies, identifying new DIPG-associated mutations with the potential for tailored therapies [6,47]. It is important to point out how the risk associated with biopsies remained virtually the same; however, a novel biologic understanding tilted the scale of risk and benefits towards the latter, as the procedure could yield valuable molecular information. It is at this time that the strikingly different histological appearance of benign and malignant tumors was associated with a new *molecular* understanding, where mutations were first seen as causative of a certain prognosis [48].

This therapeutic *potential*, however, was not readily realized, as most studies where novel chemotherapy agents (e.g., VEGF inhibitors like bevacizumab or PDGFR inhibitors like dasatinib) were used as a result of underlying, biopsy-proven genetic variations that, in theory, promised efficacy (as done in other cancer states) failed to show any significant clinical benefit [4,49].

## 4. Promise from New Clinical Trials

Despite continuous failures, fueled by an otherwise unforgiving prognosis that led to the birth of numerous funding agencies whose goal was to change it, investigative efforts continued [1]. One of the main barriers to clinical success, some felt, was represented by the blood–tumor barrier, which led to a significant decrease in drug availability within tumor parenchyma compared to the rest of the body. Thus, to achieve meaningful intratumoral concentrations of drug, dose escalation was necessary, leading to the development of numerous systemic side effects responsible for clinical trial failure [50]. The first logical solution to that issue was direct intratumoral drug delivery in the form of convection-enhanced delivery (CED). CED relies on cannula implantation into tumor parenchyma; drug delivery relies on convection which, when compared to regular diffusion, is able to achieve higher drug concentrations over a larger volume. Further, convection is mostly independent of infusate properties (i.e., it is able to achieve high drug concentration over large volumes for both small and large molecules or biologic agents), as demonstrated in pioneering experiments in the 1990s [1,51,52,53]. By virtue of its properties, CED bypasses the blood–tumor barrier and achieves high intratumoral drug concentrations, with overall low body exposure to the infusate, thus limiting systemic side effects that have caused the failure of multiple clinical trials. Albeit anecdotal evidence exists showing the feasibility of this approach, e.g., when topotecan, IL13-Pseudomonas toxin, or carboplatin were delivered in small cohorts, large studies showing the feasibility of CED were lacking [54,55,56]. Numerous preclinical efforts culminated in a dose-escalation phase I clinical trial, where convection-enhanced delivery of ^124^I-8H9 (a radioactive monoclonal antibody against the DIPG-specific B7H3 protein) was performed in 28 children with DIPG who had undergone prior radiation therapy [2,57,58]. This study showed that CED is safe, and in a subset of patients, dose escalation was well tolerated. In the study, which was not powered to assess survival, the median overall survival was 15.3 months [59]. This study further highlighted the importance of delivery monitoring, achieved here with PET imaging of the radiolabeled therapeutic agents. This work using a theranostic and similar experience using surrogate tracers reveal a variable degree of overlap of drug distribution with tumor volume, which can present a challenge for successful therapeutic deliveries [4,60].

In the realm of drug delivery, further novel approaches come, for instance, from the use of focused ultrasound. This technology is increasingly being used for focal ablation (e.g., in Parkinson’s disease or essential tremor) and relies on the direction of approximately a thousand low-intensity ultrasound beams onto a small focal point, leading to the development of a monitorable thermal injury. The procedure is performed with MRI surveillance (i.e., in the MRI scanner) so that MRI thermometry can be used to confirm lesion location and size [61,62]. In the realm of neuro-oncology, focused ultrasound had two main applications: BBB opening and focal lesioning.

Locoregional disruption of tissue cytoarchitecture at lower ultrasound intensities can reversibly open the blood–brain barrier (BBB), with clinical trials showing the safety and efficacy of this mode of BBB opening for the purported goal of increasing drug delivery to the target tissue [63]. This, coupled with selective intra-arterial drug delivery, has been shown to hold promise to increase tumor permeation [64]. Although data are still missing, clinical trials with this study design are ongoing (e.g., NCT05762419 or NCT04804709).

The second role of focused ultrasound is targeting tumor foci. Its use has been proposed for deep nodules that pose a therapeutic challenge for both open surgical intervention and laser interstitial thermal therapy (LITT), similar to what has been achieved in movement disorders [65]; thus far, however, its use remains experimental, even though numerous animal studies are emerging, showing the feasibility of such a technique [66,67,68,69].

**Figure 3:**
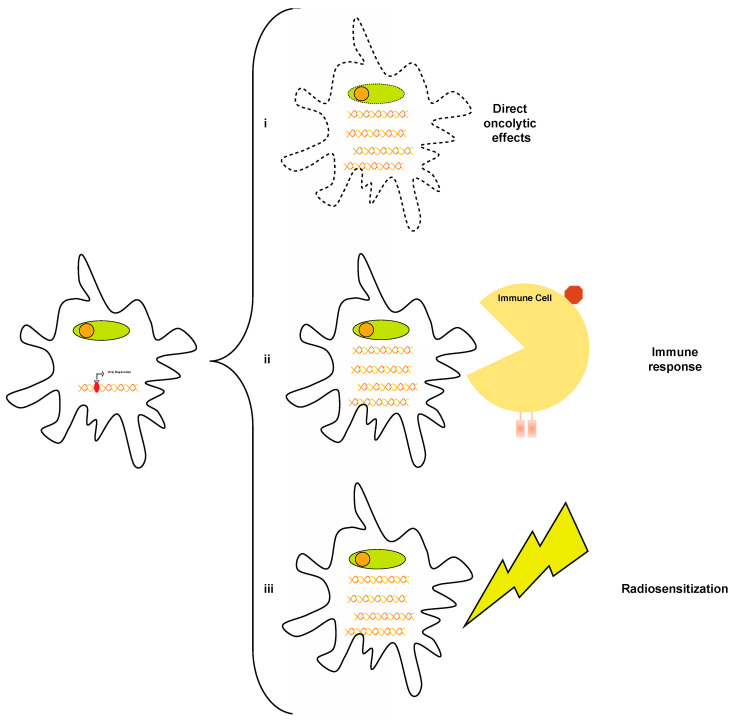
The effects of DNX-2401. The oncolytic virus DNX-2401 enters tumor cells and preferentially replicates in them. Its therapeutic activity is believed to be due to a combination of direct oncolytic effects (**i**), the elicitation of an immune response (**ii**), and radiosensitization (**iii**).

Given the failure of most classic and novel chemotherapeutic agents, other therapies have also been investigated. For instance, a recent phase I dose-escalation clinical trial assessed the safety and efficacy of intratumoral injection of DNX-2401, an oncolytic virus shown to have promise in recurrent GBM [70]. The virus is believed to work through a combination of direct oncolytic effects against DIPG cells, creating an immune response and synergistic effect with radiotherapy (Figure 3) [71,72]. In this study, investigators administered DNX-2401 following frameless stereotactic biopsy in patients with radiographic evidence of DIPG. Notably, this study relied on preoperative MRI to plan delivery and an immediate postoperative MRI to confirm it, with DNX-2401 being co-administered with gadolinium for imaging purposes. Following delivery, which did not result in grade IV or V complications in the 12 patients who were part of the study, patients underwent radiotherapy. The authors found a median progression-free survival of 10.7 months and a median overall survival of 17.8 months, along with the overall safety of their treatment approach [73].

Another promising avenue of research is represented by CAR-T therapy, which has shown clinical promise and is now FDA-approved for other diseases, such as liquid cancers (e.g., B-cell lymphomas) [74]. H3K27M-mutated DMGs are known to have high expression levels of the disialoganglioside GD2; CAR T-cells have been designed to have high affinity for this epitope and have shown great pre-clinical promise [75,76]. In a recently published early phase I clinical trial, anti-GD2 CAR T-cells were given to DMG patients (three patients with DIPG and one with a spinal DMG). In this trial, CAR T-cells intravenous administration resulted in significant tumor inflammation-associated neurotoxicities (TIANs), which the authors partially expected and for the management of which pre-enrollment protocols were in place (e.g., DIPG patients had an Ommaya reservoir placed for management of intracranial hypertension). The three patients also had a second intrathecal administration of CAR T-cells following the first intravenous one. Overall, this study showed that, with appropriate inpatient intensive care, the side effects of anti-GD2 CAR T-cell therapy are manageable; the protocol, now recruiting more patients, continues to hold promise [77].

Histone deacetylase complex (HDAC) inhibitors represent another class of therapeutics believed to have promise in DMGs. Significant preclinical evidence exists to show how DIPG and other DMGs have an altered epigenome that makes them particularly susceptible to histone regulators. For instance, the poly-HDAC inhibitor panobinostat has been shown to have significant efficacy against and specificity for DMG cell lines and animal models [78,79,80]. A recently published phase I clinical trial, however, failed to show any significant clinical benefit in young patients with DIPG or other DMGs because of the development of significant systemic dose-limiting toxicities (e.g., myelosuppression and thrombocytopenia) that made dose escalation not possible [81]. CED rose as a possible solution to this issue: repeated CED of MTX110 (a soluble HDAC inhibitor) was shown to be tolerable and achieved a median overall survival of 26.1 months in seven children with previously radiated DIPG [60].

## 5. Current Standard of Care

Since their first description by Dr. Harvey Cushing more than a century ago, little has changed for the majority of patients with DMGs: with no surgical option available and a lack of systemic therapies, patients slowly succumb after a short-lived radiation-induced improvement. We are, however, on the edge of a revolution, with the first small set of clinical trials starting to show benefit in a selected patient population.

**Figure 4:**
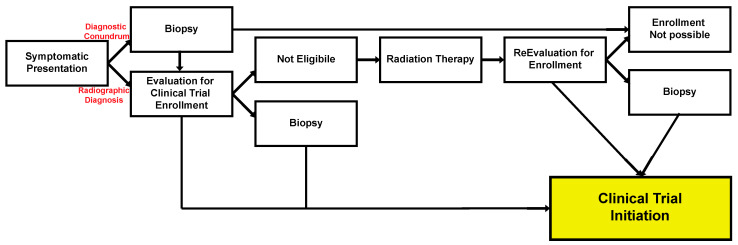
Current standard of practice in DMG patients. Given the importance of clinical trial enrollment due to the lack of effective systemic therapies, that should be the goal for DMG patients. Radiation therapy should be considered early on for symptomatic management. Depending on the clinical course, the patient can and should be re-evaluated for enrollment as their disease progresses. Biopsy remains available in cases of diagnostic uncertainty or if required for trial purposes.

To continue this revolution and provide DMG patients with a therapeutic option, albeit feeble as it may be, a new treatment paradigm has to be in place. Upon presentation in patients with a suspected DMG, evaluation for possible enrollment in a clinical trial should be the first step unless radiation therapy is urgently needed for symptom amelioration. If the patient is eligible for participation and willing to, enrollment should follow, either before or after biopsy (if needed for clinical trial purposes or if at a diagnostic conundrum) and either before or after conventional external beam radiation therapy, depending on patient symptomatology and specific clinical trial inclusion criteria. If enrollment is not possible at presentation (e.g., the patient has too low of a functional score), then conventional radiation therapy should follow, at which point the patient should be re-evaluated for clinical trial enrollment (and, again, biopsy should be considered if needed). Notably, given the low risk now associated with biopsies and the requirement of many trials to have a specific DMG histology or mutation pattern, a provider should consider this step early to hasten enrollment. Overall, given the promise of some recent clinical trials, this should be the goal for patients with DMGs (Figure 4). Symptomatic management in the hands of experienced practitioners should continue through the patients’ journeys.

Further promise comes from the realm of liquid biopsies; in their simplest forms, they are able to detect tumor antigens in peripheral blood. Different variations of liquid biopsies exist, depending on whether they detect circulating nucleic acids, tumor DNA, or tumor cells [82]. Recent efforts were aimed at the tracking of mutations in the cerebrospinal fluid of patients with known CNS malignancies. When tumor DNA was found in the CSF, it was found to have numerous mutations shared with the parent tumor (as determined by biopsy), while others (e.g., growth factor receptors) showed significant evolution [83,84,85]. Albeit not part of clinical practice yet, liquid biopsies bring forward the hope of their lack of invasiveness in DMGs not only for diagnosis but also for response tracking following therapy, as they have done in other solid malignancies [86,87,88]. This is particularly the case given the role of pseudoprogression, i.e., radiographic changes in tumor appearance due to treatment rather than actual tumor progression. This is challenging for DMGs, where treatment-related changes (following chemotherapy or radiation) often present as edema with T2 shortening on MRI or FLAIR imaging, similar to actual tumor growth. Some have advocated the use of T2/FLAIR mismatch (an imaging finding where a lesion is characterized by a strong T2 shortening but appears hypointense on FLAIR imaging) as a tool for response monitoring, but this needs further clinical validation [27]. Novel algorithms are still being developed to optimize clinical protocols; however, early clinical applications are starting to emerge with significant clinical promise [89,90,91].

Overall, DMGs are one of the most complex and challenging therapeutic entities, characterized by a complex genetic and epigenetic profile, unforgiving locations that take them out of the surgical realm, and a rapid clinical course often leading to precipitous clinical deterioration. In recent years, however, a novel biological understanding has changed the clinical landscape of this diagnosis, and it is now starting to shape new therapeutic strategies that will undoubtedly lead to significant clinical improvements pioneered by novel and exciting clinical trials.

## 6. Conclusions

Diffuse midline gliomas represent a challenging pathological entity. Despite significant advances in neurosurgical oncology, they remain virtually inoperable, with prognosis ameliorated by palliative radiation only. This is due, in part, to their location, centered around numerous deep nuclei key in motor processing and essential for biological functions (e.g., breathing). In recent years, however, a novel biological understanding of these tumors, stemming in part from increased tissue availability made possible by ever-safer biopsies, has led to a therapeutic revolution. Numerous targeted clinical trials are now ongoing, and with new data showing increased feasibility of these novel approaches, hope is finally on the horizon.
